# Effects of methamphetamine on signalled probability in rats using concurrent chains

**DOI:** 10.1007/s00213-026-07006-x

**Published:** 2026-01-23

**Authors:** Rebecca Rose Hazel Bodeker, Randolph C. Grace

**Affiliations:** Department of Psychology Speech, and Hearing, University of Canterbury Christchurch, 20 Kirkwood Avenue, Upper Riccarton, Christchurch, 8041 New Zealand

**Keywords:** Methamphetamine, Sensitivity to probability, Signalling effect, Concurrent chains, Rats

## Abstract

Pigeons show a suboptimal choice for probabilistic reinforcement in the presence of informative stimuli, which has been proposed as an animal model of gambling. However, this behaviour has been difficult to replicate in rats. In our study, rats responded on alternatives that provided 50% or 100% reinforcement with half of the sessions reliably signalled and half un-signalled. Contrary to expectation, rats preferred the optimal alternative to a greater extent in the signalled sessions compared to the un-signalled sessions. Acute methamphetamine increased preference for the 50% alternative exclusively in signalled sessions. These results suggest that rats, unlike pigeons, are able to use the information provided by the stimulus correlated with non-reinforcement to shift preference away from the suboptimal alternative, and that the influence of methamphetamine on rats’ probabilistic choice is context dependent.

**Supplementary Information:**

The online version contains supplementary material available at https://doi.org/10.1007/s00213-026-07006-x.

## Introduction

Gambling is an often studied scenario of impulsivity and probabilistic choice (Alessi and Petry 2003; Castellani and Rugle 1995; Rivalan et al. 2009; Zentall and Stagner 2011). It is estimated that 1.41% of adults globally exhibit problem gambling (Tran et al. 2024). People that engage in gambling behaviour to the point of addiction are likely to exhibit high levels of dysfunctional impulsivity that predispose them to seek immediate rewards despite almost inevitable serious consequences (Ioannidis et al. 2019; Nower and Blaszczynski 2006). Gambling is strongly associated with other forms of addiction, most notably, substance misuse. Lesieur and Blume (1993) as cited in Petry (2001) suggest that up to 50% of problem gamblers have a co-morbid substance use disorder, which is often related to stimulants. Our study will investigate the effect of methamphetamine on rat’s choice behaviours in an analogue gambling task.

Gambling-related decision making can be modelled in animal studies. One of the most addictive and frequently studied forms of gambling, including with animal models, is slot machine gambling also known as electronic gaming machines. Murch and Clark (2016) explain that the rapid pace of play and the variable ratio of reinforcement increase the addictive potential of slot machines. They also suggest that the sensory output of the machines which include flashing lights, catchy music and increasing ‘jackpot’ totals is sufficient to cause gamblers to continue to play even when they are losing money. Some of these features of slot machine gambling can be incorporated into animal sensitivity to probability procedures resulting in an outcome termed the ‘signalling effect’ where animals behave suboptimally in the presence of a signal for reinforcement.

Zentall and Stagner (2011) studied probabilistic choice in pigeons in a model of gambling. In their first experiment, pigeons preferred the suboptimal option when the food and no-food outcomes were differentially signalled. The authors suggested that similar processes may govern human gambling as players are informed of wins (and losses) before they receive the money. They also suggest that a contrast in expectation may influence behaviour in both pigeons and human gamblers where the low probability of winning creates a large amount of positive contrast when it is won, whereas when the high probability loss occurs, the negative contrast is small because it is expected. This creates a net positive contrast for responding on the suboptimal alternative which drives further gambling.

However, not all animals respond suboptimally when outcomes are signalled. When rats have completed an equivalent procedure that would result in suboptimal choice in pigeons, rats behaved optimally (Mazur 2005; Orduña & Alba, 2019; Roper and Baldwin 2004; Trujano et al. 2016; Trujano and Orduña 2015). In Trujano and Orduña’s (2015) study, rats preferred the optimal alternative where the signals were not correlated with the outcome over the suboptimal where signals were predictive.

However, Cunningham and Shahan (2019) showed that rats’ preference for the optimal alternative depended on the overall delay to reinforcement. For nine out of ten rats, responding on the suboptimal alternative with predictive signalling increased as the terminal link lengthened, with most subjects shifting their choice from the optimal non-predictive alternative to the suboptimal predictive alternative at the 30 s terminal link. The authors suggested that rats, like pigeons will forgo food for information when the duration of the correlated signal is sufficiently long.

Few studies have examined the influence of stimulant drugs on rats’ choice in signalled procedures. Cardinal et al. (2000) examined how rats would respond in a delay discounting task if the delay was signalled alongside amphetamine administration. Amphetamine increased preference for the large delayed lever, but only at the lowest dose compared to baseline. The authors suggested that amphetamine exaggerated the conditioned reinforcing potential of the cue which increased preference for the delayed reinforcer.

The current research used a novel task to assess the effects of methamphetamine on signalled probabilistic choice. Prior studies on the signalling effect have used procedures in which a single response is required on each trial, with a mixture of free and fixed choice trials (in which only a single alternative is presented) arranged to ensure exposure to both outcomes (Zentall and Stagner 2011; Trujano and Orduña 2015; Cunningham & Shahan, 2019). We used a free-operant task in which rats made multiple responses during an extended choice period to produce access to outcomes associated with a 100% or 50% chance of food reward, and assessed preference under both signalled and non-signalled conditions. After baseline training, saline and three increasing acute doses of methamphetamine were scheduled followed by chronic administration (12 consecutive sessions). We expected to observe a signalling effect in baseline where preference for the 50% alternative would be greater under signalled than non-signalled conditions. With increasing doses of methamphetamine, we predicted that preference for the 50% alternative would increase under both signalled and non-signalled conditions.

## Method

Previous research has shown that animals can learn to quickly adjust their choice behavior to unpredictable changes in reinforcement contingencies across sessions (e.g., Grace et al. 2003; McLean et al. 2018). We used this method to assess rats’ preference for probabilistic reinforcement in a concurrent-chains task. On each trial, rats responded during a choice phase (‘initial links’) to produce access to one of two alternative schedules that delivered reinforcement on 50% or 100% of trials (‘terminal links’). In each block of four sessions, we pseudo-randomly varied both the left/right location of the 50% and 100% alternatives and whether the outcomes (food or no food) of the 50% alternative were reliably signalled.

**Fig. 1:**
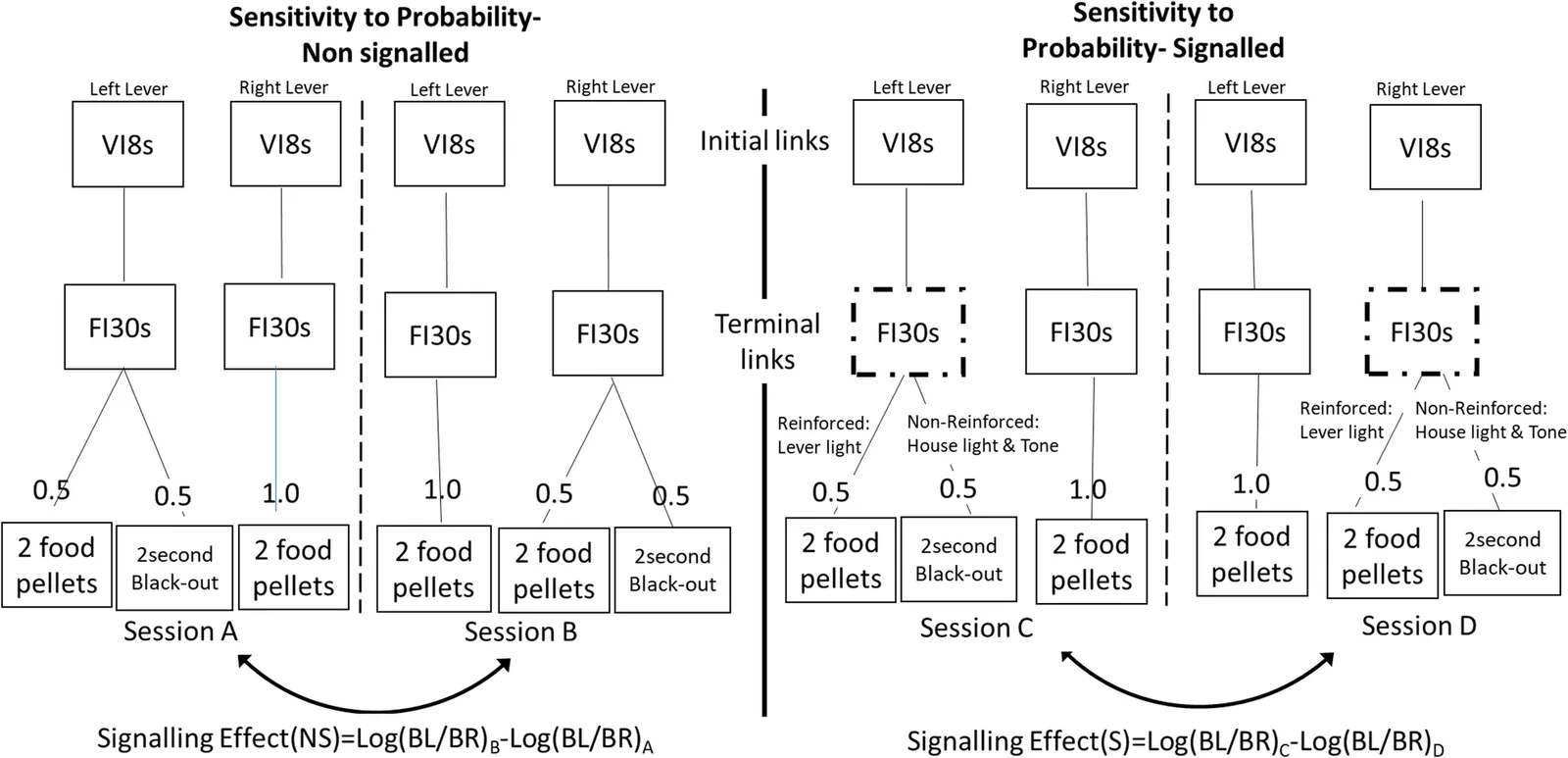
Illustration of four session types (A, B, C, D) that were arranged pseudo-randomly to assess sensitivity to probability (preference for 100% over 50% alternative) under conditions in which the outcomes for the 50% alternative were Non-signalled or Signalled. See text for more explanation

The four types of sessions are shown in Fig. 1. For Sessions A and B, the outcomes for the 50% alternative (2 food pellets or a 2-s blackout) were non-signalled and the 100% alternative was associated with the right and left lever, respectively. Thus, comparing the (log) response ratio between Sessions B and A gives an estimate of choice for the 100% over 50% alternative under non-signalled conditions. Sessions C and D were similar except that the 50% outcomes were signalled (lever light was illuminated on food trials; flashing houselight and tone on no-food trials), so that comparing the (log) response ratio between sessions D and C gives an estimate of choice for the 100% alternative under signalled conditions.

### Subjects

Twelve black hooded/PVG (Piebald Virol Glaxo) male rats were used in this study. They were bred in the University of Canterbury Animal Lab and were a minimum of 100 days old when training commenced. All experimental procedures were approved by the University of Canterbury Animal Ethics Committee, Reference: 2018/18R. The subjects were maintained at 85% of their free feeding weight throughout the training and experimental phase with a combination of 45 mg pellets (Bio-Serve Dustless Precision Pellets, Sucrose, Chocolate Flavour), used as the reinforcer in the testing chambers, and supplemental food pellets (LabDiet Prolab RMH 1800) given at the conclusion of the session. Rats were housed in groups of four with free access to water except when performing the experiment and had shredded paper and wood pellets for nesting substrate. Humidity and temperature were controlled, ventilation ensured adequate air exchange and the 12 h light/dark cycle turned the lights on at 0700 h. Methamphetamine solution was administered interperitoneally. At the conclusion of the experiment, the subjects were euthanised by qualified lab technicians according to American Veterinary Medical Association guidelines.

### Apparatus

Subjects completed the daily testing procedure in ‘MED-008-CT-B2 Basic Self Administration Package with Standard Chamber and Retractable Levers for Rat’ chambers. The chamber measured 29.53 cm x 24.84 cm x 18.67 cm and was surrounded by a sound attenuating wooden box. All sides of the chamber were made of plexiglass and subjects stood on stainless steel bars raised 7 cm from the bottom of the chamber. They responded on the right side of the chamber on two 5 cm long retractable levers that were placed 10 cm above the bars and 13 cm apart. Lever lights were located in the centre of and 5 cm above each lever. A house light was located on the left side of the roof of the chamber. Reinforcement was delivered via a modular pellet dispenser that dropped 45 mg chocolate flavoured pellets into the Trough Type Pellet Receptacle located between the two levers and 3 cm above the floor. Data was collected by the MED-PC for Windows programme, Version IV. Statistical analysis was completed with Jamovi 2.3.16 software which uses the LME4 R package. Jamovi uses an unstructured co-variance matrix and restricted maximum likelihood for parameter estimation. The GAMLj module was installed to perform mixed model analysis.

### Training

Rats were first trained to collect a food pellet from the receptacle regardless of lever interaction. This was followed by several sessions of auto-shaping training. After a 30-s intertrial interval, both levers were extended and a food pellet reinforcer was delivered after 8 s, unless a response to either lever occurred in which the reinforcer was delivered immediately. Sessions ended after 45 reinforcers had been delivered. Manual shaping was given as necessary until all subjects could reliably respond on both levers and collect all reinforcement.

Then, sessions with 8 s initial links and 5 s terminal links were presented so that the rats learned to respond in the concurrent-chains task until they reliably completed 72 trials and received two pellets per reinforcement. Signalled sessions were introduced next along with the location of the 50% and 100% outcomes changing unpredictably between sessions. Finally, the terminal link length was extended to 30 s to ensure adequate exposure to the signal while the number of trials per session was reduced to 54 to limit session length to 60 min and the signal for reinforcement was restricted to the 0.5 alternative on signalled sessions.

### Procedure

The task was a concurrent-chains procedure. Each trial commenced with a VI8s initial link in which both left and right levers were extended, and the lights above both levers and the house light were illuminated. Responses on both levers were recorded to measure the rats’ preference (i.e., which of the two schedules they would prefer to progress to the terminal link) until the VI8s timer elapsed. A further lever press on the option to which the reinforcer was assigned initiated the terminal link, retracted the non-active lever, and extinguished the light above that lever.

If the session was non-signalled, the lever light above the remaining active lever was turned off, responses were recorded, and reinforcement was always delivered on the 100% option after the 30 s had elapsed and a final press was made to the active lever. Reinforcement was delivered half the time on the 50% option after the FI30s terminal link and a final response to the active lever. The remaining half of the sessions on the 50% option were not reinforced, and a 2 s blackout occurred following the final lever press, after which the next trial was started.

If the session was signalled, the light above the 50% lever flashed (0.25s on and 0.25s off), during the FI30s terminal link if reinforcement would be delivered, whereas the house light flashed (0.25s on and 0.25s off) accompanied by an intermittent tone every 0.5s on non-reinforcement trials. A final press on the 50% lever initiated pellet delivery or 2 s blackout respectively. We did not counterbalance assignment of lever light vs. houselight/tone so that the location of the signal for reinforcement was always associated with the lever that produced it considering that the location of the active lever changed unpredictably. The 100% alternative always delivered reinforcement in the signalled sessions following a final press of the lever, but no stimuli were presented during the terminal link. Responses on the active lever were recorded during the terminal link in order to determine if the rats interpreted the signals correctly. If food was delivered, the receptacle light was illuminated, and the dispenser delivered 2 pellets. If subjects failed to respond within 5 s of the conclusion of the terminal link, a 2 s blackout occurred, and the next trial started. The amount of reinforcement was held constant across session type and probability, with the left lever providing two pellets of reinforcement and the right lever providing two pellets of reinforcement. Following reinforcer delivery, the receptacle light was extinguished and another initial link commenced. This process was repeated until 54 trials had been completed or an hour elapsed, whichever occurred first.

Table 1 shows the phases of training across the experiment. Rats completed 30 sessions of baseline training, followed by the acute drug administration phase, chronic drug administration phase, and finally a return to baseline.

**Table 1:** Order of experimental phases

Phase	Stages	No. of Sessions
Baseline	Signalled and Non-Signalled Sensitivity to Probability	30
Acute Drug	Two non-drug days followed by one day of 0.0 mg/kg, 0.5 mg/kg, 1.0 mg/kg or 2.0 mg/kg methamphetamine	96
Chronic Drug	4.0 mg/kg 23 h prior to session start	12
Re-establish Baseline	Signalled and Non-Signalled Sensitivity to Probability	16

In the acute drug phase, a drug administration session was followed by two non-drug administration sessions. Rats completed a session each day regardless of the presence or absence of methamphetamine. The sequencing of sessions was arranged from four possible session types, two which assessed non-signalled probabilistic choice (A and B in Fig. 1), and two which assessed signalled probabilistic choice (C and D in Fig. 1).

In the acute phase, sessions were arranged so that the effect to be tested under drug administration was first tested in non-drug administration sessions. For example, if a signalled sensitivity to probability effect was to be tested, the two non-drug sessions preceding the drug session would also measure signalled sensitivity to probability. The first drug administration session for signalled probabilistic choice (C or D) was then followed by two non-drug days in which non-signalled probabilistic choice would be assessed (A and B). The second of the signalled sessions would then be run, allowing an estimate of sensitivity to probability to be obtained under drug administration. This procedure would then be repeated to obtain a non-drug and drug measure of the remaining effect; in this example, non-signalled sensitivity to probability. Thus, a block of twelve sessions was required to obtain estimates of signalled and non-signalled sensitivity to probability under drug administration. Two such blocks were run for each drug dose.

A saline control and three drug doses were administered in the acute phase half an hour prior to the start of a session. In order to examine the effects of the receipt of an injection, a saline only dose was administered first (0.0 mg/kg). This was followed by 0.5 mg/kg of methamphetamine solution, then 1.0 mg/kg of methamphetamine solution and finally, 2.0 mg/kg of methamphetamine solution (Richards et al. 1999). All solutions were administered intraperitoneally, at a volume of 1 ml/kg. Each drug administration day was followed by two non-drug administration days. Six days were required to measure a single effect, two signalled and two non-signalled probability sensitivities were obtained per dose with four doses measured, resulting in 96 sessions for the acute methamphetamine phase.

The chronic phase consisted of twelve injections administered 23 h before the start of a session with one injection given per day for 12 subsequent days (Richards et al. 1999). Three signalled and three non-signalled probability sensitivities were obtained in this phase. These doses attempted to replicate acute and chronic methamphetamine use to model both the use of acute stimulant doses in clinical populations, increasing use over time, and chronic use in substance abuse users.

The final phase of the experiment re-established a baseline measurement of signalled and non-signalled sensitivity to probability. No drug was administered in this phase. This phase commenced immediately after the completion of the chronic phase and ran for 16 sessions allowing for four signalled and four non-signalled probability sensitivities to be measured.

### Statistical analysis

Analyses were based on a generalised matching model:

1 $$\log\frac{B_L}{B_R}=\log b+a_P\log\frac{1/P_L}{1/P_R}$$

where *B* is response rate, *P* is probability of reinforcer receipt, and subscripts *L* and *R* indicate the choice alternatives. There are two parameters: sensitivity to probability (non-signalled or signalled) and bias, *log b.* By examining differences in log response ratios between sessions in which probability was non-signalled; A&B, or signalled; C&D, we were able to obtain estimates of non-signalled and signalled sensitivity to probability in each of four sessions. Higher sensitivity to probability indicated greater preference for 100% alternative and lower sensitivity to probability indicated preference for the uncertain 50% alternative.

We conducted a lag regression analysis using the final 20 sessions of baseline training. This analysis used a model with predictors for both current and prior session contingencies, and was applied to successive blocks of training within sessions so that we could assess the acquisition of choice within sessions:

2 $$\:log\frac{{B}_{L0}}{{B}_{R0}}=\mathrm{log}b+{p}_{0}log\frac{1/{P}_{L0}}{1/{P}_{R0}}+{p}_{1}log\frac{1/{P}_{L1}}{1/{P}_{R1}}+{s}_{0}+{s}_{1}$$

In Eq. 2, *B* is response rate, *P* is probability of reinforcement, and subscripts indicate the left/right lever (*L*, *R*) and lag (0 = current session; 1 = prior session). There are five parameters: Bias (log *b*), sensitivity to probability (*p*; lag 0 and 1), and sensitivity to signal (*s*; lag 0 and 1).

## Results

### Baseline

**Fig. 2:**
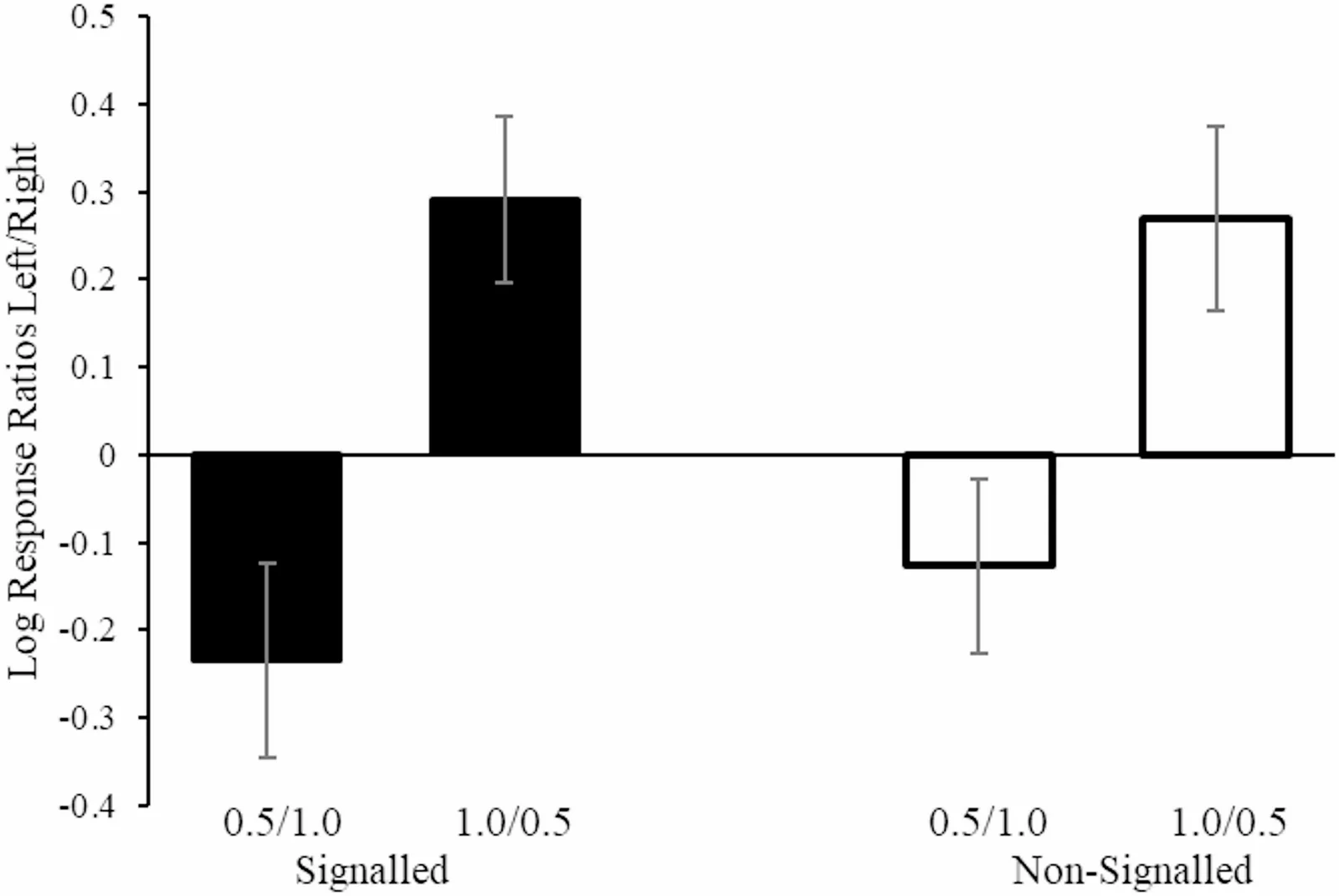
Average Baseline Log response ratios of four possible session types, 0.5/1.0 Signalled, 1.0/0.5 Signalled, 0.5/1.0 Non-Signalled, 1.0/0.5 Non-Signalled. Bars indicate +/- 1 standard error (SE)

Figure 2 shows the average log response ratios (left/right) for the four session types across the last 20 sessions of baseline training. To characterise the baseline results, the effect sizes of the signalled and non-signalled sessions were compared. A 2 × 2 Repeated Measures ANOVA found a significant effect of Signalled/Non-Signalled, *F*(1,11) = 5.057, *p* <.05, η²_p_=.315, Polarity, *F*(1,11) = 224.524, *p* <.05, η²_p_=.953, and a Signalled/Non-Signalled x Polarity interaction, *F*(1,11) = 6.180, *p* <.05, η²_p_=.360. Mean sensitivity to probability was Signalled 0.5/1= −0.235, Signalled 1/0.5 = 0.291, Non-Signalled 0.5/1= −0.127, Non-Signalled 1/0.5 = 0.269. A t-test demonstrated that the difference between the two signalled session types was significantly greater than the difference between the two non-signalled session types, *t*(11) = 2.486, *p* <.05. Therefore, baseline Signalled Sensitivity to Probability was significantly greater than Baseline Non-Signalled Sensitivity to Probability, meaning that subjects preferred the certain option to a greater extent in the presence of the signal than in the absence of the signal.

**Fig. 3:**
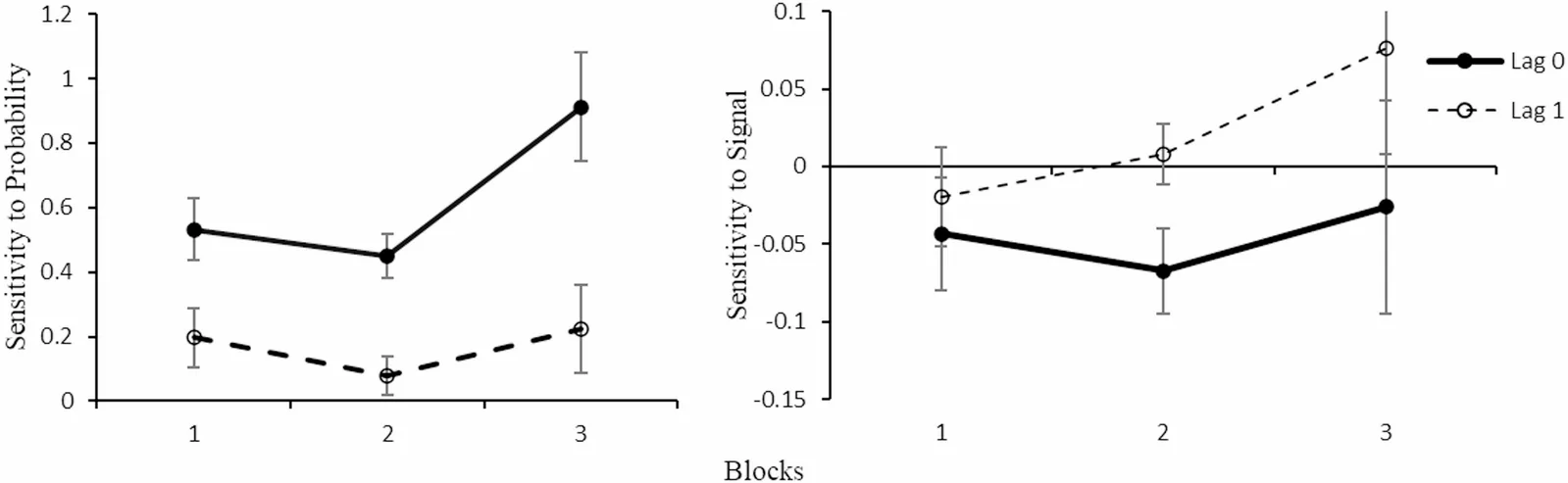
Lag Regression Analysis Results displaying average Sensitivity to Probability (Left) and Sensitivity to Signal (Right) across 18 -trial blocks of a session. Subjects 201–212. Bars indicate +/- 1 standard error (SE)

Figure 3 shows best-fitting parameter estimates for Eq. 2 which were obtained for each of the three session blocks for individual rats (overall average R^2^ = 0.315). A linear mixed effects model with lag and block as fixed effects and a random intercept was applied to the sensitivity to probability data. The intra-class correlation was significant, *r*_ICC_ = 0.269, *p* <.05, showing that the random effect accounted for 26.9% of the variance. There was a significant effect of lag, *F*(1,55) = 36.728, *p* <.05 showing that sensitivity to probability was greater for Lag0 (*M* = 0.631; 95% CI = 0.466, 0.796) than Lag1 (*M* = 0.166, 95% CI = 0.001,0.331). There was a significant effect of Block, *F*(2,55) = 5.426, *p* <.05. Analysis of simple effects showed that for Lag 0, sensitivity to probability was significantly lower in Block 1, (*M* = 0.531, 95% CI = 0.310,0.752) and Block 2, (*M* = 0.450, 95% CI = 0.230,0.670) when compared to Block 3, (*M* = 0.912, 95% CI = 0.691,1.133). There was a significant linear component, *t*(2,55) = 2.863, *p* <.05, and a quadratic component, *t*(2,55)=−2.361, *p* < 05. For Lag 1, there no significant differences between Block 1 and the remaining Blocks, and confidence intervals showed that none were significantly different from zero. Thus, choice responding was controlled by the probabilities in the current session with no systematic effect of the prior session.

A linear mixed effects model with lag and block as fixed effects and a random intercept was applied to the sensitivity to signal data. The intra-class correlation was not significant, *r*_ICC_ = 0.174, *p* >.05. The effect of lag approached significance, *F*(1,55) = 3.790, *p* =.057 showing a trend for sensitivity to signal to be smaller for Lag0 (*M* = −0.046; 95% CI= −0.110, 0.019) than Lag1 (*M* = 0.021, 95% CI= −0.043,0.086. None of the other effects were significant.

### Acute

**Fig. 4:**
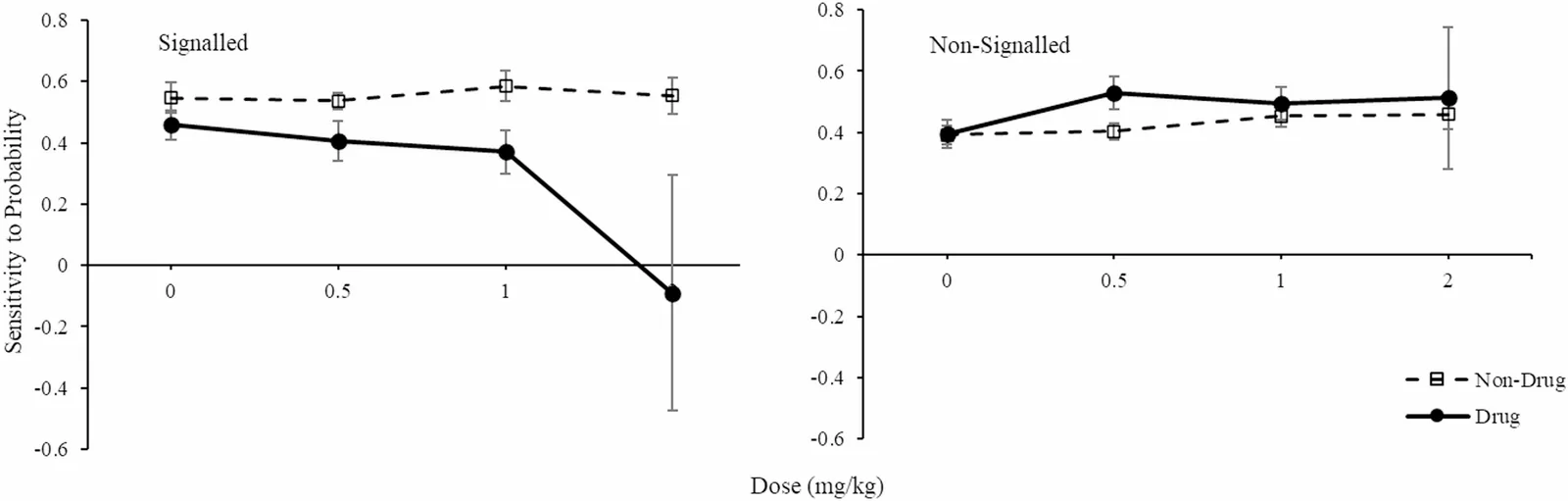
Average Signalled Sensitivity to Probability (Left) and Non-Signalled Sensitivity to Probability (Right). for Acute Non-Drug sessions and 0, 0.5, 1.0 and 2.0 mg/kg methamphetamine/Drug sessions. Subjects 201–212. Bars indicate +/- 1 standard error (SE)

Figure 4 displays the averaged Signalled Probability (left), and Non-Signalled Probability Effects (right) across the four drug doses for both the non-drug and drug sessions of the acute phase. A linear mixed effect model with Signalled/Non-Signalled, Dose and Drug/Non-Drug as fixed effects and a random intercept was applied to the data. The intra-class correlation was significant, *r*_ICC_=0.091, *p* <.05, showing that the random effect accounted for 0.91% of the variance. There was a significant effect of Drug/Non-Drug, *F*(1,160.477) = 5.838, *p* <.05 showing that sensitivity to probability was greater for Non-Drug (*M* = 0.491; 95% CI = 0.404,0.579) than Drug (*M* = 0.382, 95% CI = 0.292,0.471) sessions. There was a significant Drug/Non-Drug x Signalled/Non-Signalled interaction, *F*(1,160.477) = 13.374, *p* <.05. Analysis of simple effects showed that the Signalled No Drug Probability Effect Size (*M* = 0.556; 95% CI = 0.450,0.661) was significantly greater than the Signalled Drug Probability Effect Size (*M* = 0.280; 95% CI = 0.168,0.392). There was no significant difference between the Non-Signalled No Drug and Non-Signalled Drug Probability Effect Sizes. There was a significant Dose x Signalled/Non-Signalled interaction, *F*(3,160.445) = 2.929, *p* <.05. Analysis of simple effects showed that the 2.0 mg Non-Signalled Probability Effect Size (*M* = 0.486; 95% CI = 0.350,0.623) was significantly greater than the 2.0 mg Signalled Probability Effect Size (*M* = 0.219; 95% CI = 0.063,0.374), and that the 0.0 mg Signalled Probability Effect Size (*M* = 0.502; 95% CI = 0.366,0.639), was significantly greater than the 2.0 mg Signalled Probability Effect Size, (*M* = 0.219; 95% CI = 0.063,0.374). There was no significant difference between Dose and the Non-Signalled Probability Effect Sizes. The linear component of the Drug Signalled Probability Effect Size was significant, *t*(3,162.058)=−3.835, *p* <.05, as was the quadratic component, *t*(3,161.351)=−2.251, *p* <.05. None of the other effects were significant, and all other *p* values > 0.1.

In the No Drug sessions, sensitivity to probability was greater when lack of reinforcement was Signalled than when it was Non-Signalled. When it was Signalled, sensitivity to probability was greater for the No Drug sessions than the Drug sessions and sensitivity in the Drug sessions dose dependently decreased. There was no significant difference between the Drug and No Drug sessions in the Non-Signalled sessions.

### Chronic

**Fig. 5:**
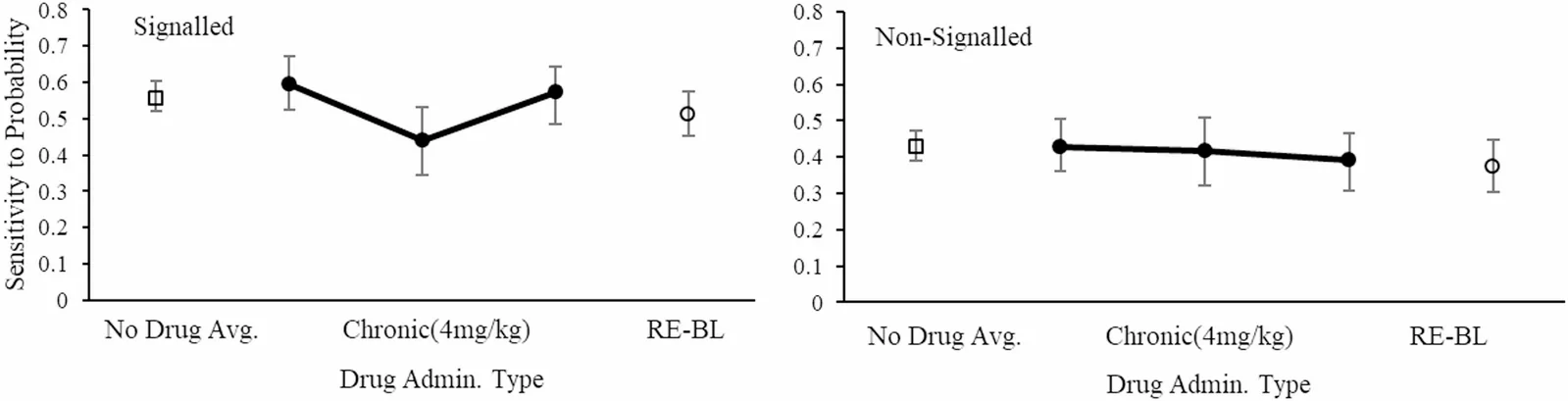
Average No Drug Sensitivity to Probability Effect Size for Acute Phase, three average Chronic 4 mg/kg methamphetamine Sensitivity to Probability Effect Sizes and average Re-Establish Baseline Sensitivity to Probability Effect Size for Signalled and Non-Signalled Sessions. Subjects 201–212. Bars indicate +/- 1 standard error (SE)

Figure 5 displays the average no drug days from the acute drug administration phase, the three chronic phase effect sizes where 4.0 mg/kg of methamphetamine was administered 23 h prior to the start of the session, and the average re-establish baseline effect sizes for both signalled and non-signalled sessions. A linear mixed effects model with Signalled/Non-Signalled, and Drug Administration Type as fixed effects and a random intercept was applied to the data. The intra-class correlation was significant, *r*_ICC_=0.281, *p* <.05, showing that the random effect accounted for 28.1% of the variance. There was a significant effect of Signalled/Non-Signalled, *F*(1,99) = 10.424, *p* <.05. Estimated marginal means showed that the Signalled Sensitivity to Probability, (*M* = 0.532, 95% CI = 0.433, 0.631) was significantly greater than the Non-Signalled Sensitivity to Probability, (*M* = 0.408, 95% CI = 0.308, 0.507). Simple Effects revealed that the third chronic dose Signalled Sensitivity to Probability (*M* = 0.572, 95% CI = 0.428, 0.715), was significantly greater than the third chronic dose Non-Signalled Sensitivity to Probability, (*M* = 0.393, 95% CI = 0.249, 0.536). There were no other significant effects, and all other *p* values > 0.2.

The Sensitivity to Probability for Signalled and Non-Signalled was not influenced by the chronic drug administration when compared to both the acute no-drug average and the re-establish baseline average. However, Sensitivity to Probability was greater in the signalled sessions than the Non-Signalled sessions. These results show that in the no drug acute sessions and chronic administration sessions, sensitivity to probability was greater when the outcome of the 50% alternative was signalled compared to when it was not. Acute methamphetamine dose dependently reduced sensitivity to probability only in sessions where the outcome of the 50% alternative was signalled in the terminal link.

### Terminal link response rates

**Fig. 6:**
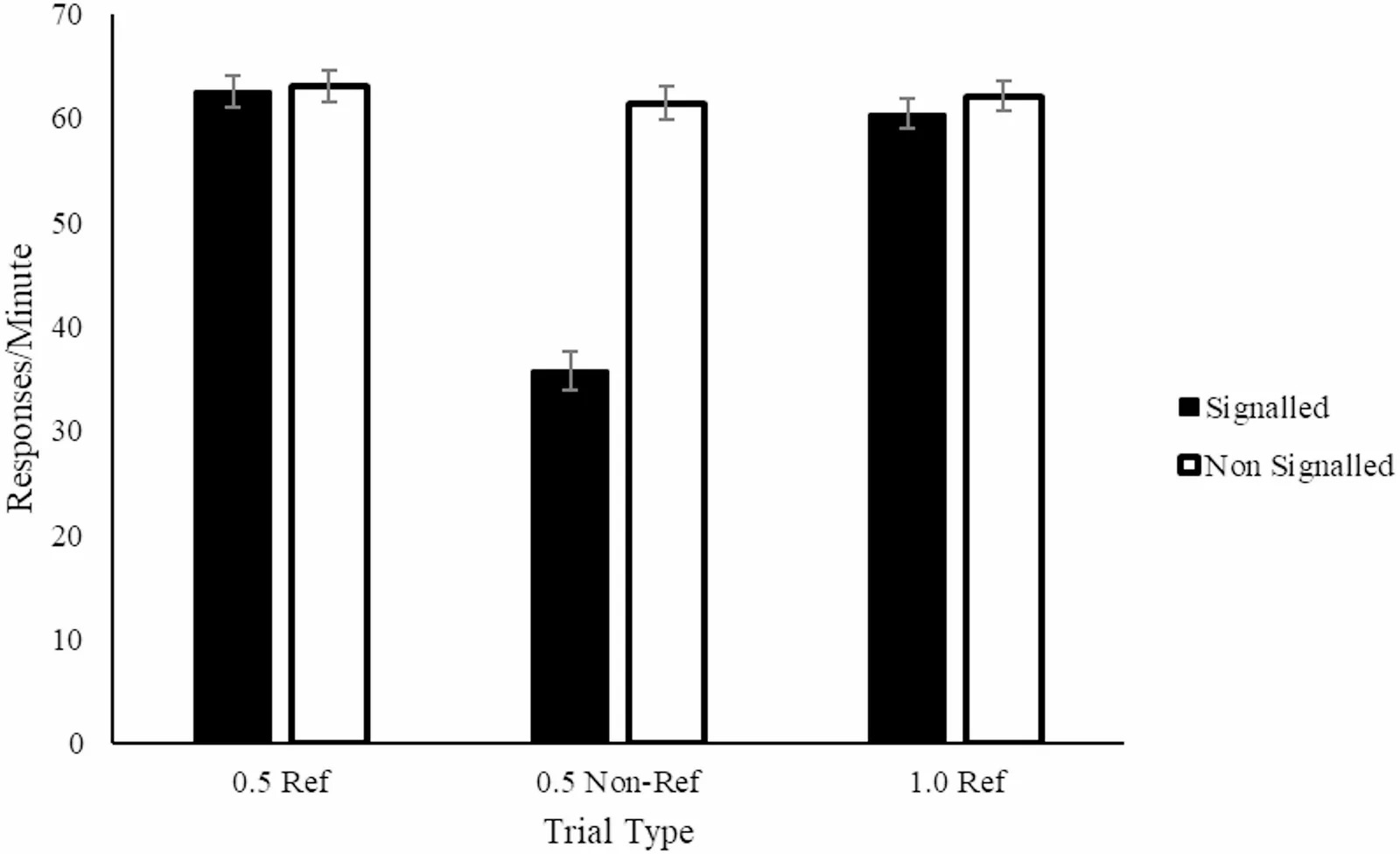
Average Baseline and Re-Establish Baseline Terminal link response rates by trial type and signalled/non-signalled. Subjects 201–212. Bars indicate +/- 1 standard error (SE)

In order to determine that rats were able to discriminate the flashing house light and tone signal during the terminal links for non-reinforcement, we calculated the terminal link response rates according to the three possible trial types within each session; 0.5 with reinforcement, 0.5 without reinforcement and 1.0 which was always reinforced. In half of the sessions, lack of reinforcement was signalled, and in the remaining half, it was non-signalled. Average response rates are shown in Fig. 6 and suggest that rats responded at lower rates during the flashing house light and tone. A linear mixed effects model with Signalled/Non-Signalled, Baseline/Re-Establish Baseline and Trial Type as fixed effects and a random intercept was applied to the data. The intra-class correlation was significant, *r*_ICC_=0.466, *p* <.05, showing that the random effect accounted for 46.6% of the variance. There was a significant effect of Signalled/Non-Signalled. *F*(1,1273) = 192.734, *p* <.05, with simple effects demonstrating that Non-Signalled sessions (*M* = 62.235, 95% CI = 55.086, 689.383), had a significantly greater terminal link response rate compared to Signalled sessions (*M* = 52.966, 95% CI = 45.817, 60.114). There was a significant effect of Baseline/Re-Establish Baseline *F*(1,1273) = 28.410, *p* <.05, where Baseline sessions (*M* = 59.380, 95% CI = 52.235, 66.524) had a significantly greater terminal link response rate than re-establish baseline (*M* = 55.821, 95% CI = 48.668, 62.974). There was a significant effect of Trial Type, *F*(2,1273) = 182.568, *p* <.05, and a significant Signalled/Non-Signalled x Trial Type interaction, *F*(2,1273) = 149.570, *p* <.05. Simple Effects revealed that in Baseline and Re-Establish Baseline, Signalled 0.5 Non-Reinforced (*M* = 35.835, 95% CI = 28.606, 43.064) was significantly smaller than Non-Signalled 0.5 Non-Reinforced, (*M* = 61.419, 95% CI = 54.190, 68.649), both Signalled and Non-Signalled 0.5 Reinforced, (*M* = 62.647, 95% CI = 55.418, 69.876), (*M* = 63.140, 95% CI = 55.911, 70.369), and both Signalled and Non-Signalled 1.0 Reinforced, (*M* = 60.415, 95% CI = 53.186, 67.644), (*M* = 62.145, 95% CI = 54.916, 69.374).

These results demonstrate that when the lack of upcoming reinforcement was signalled, subjects significantly decreased their response rates both prior to and following drug administration, compared to when lack of reinforcement was not signalled or was forthcoming, demonstrating that subjects could discriminate the signals and changed their behaviour accordingly.

### Discrimination ratio

**Fig. 7:**
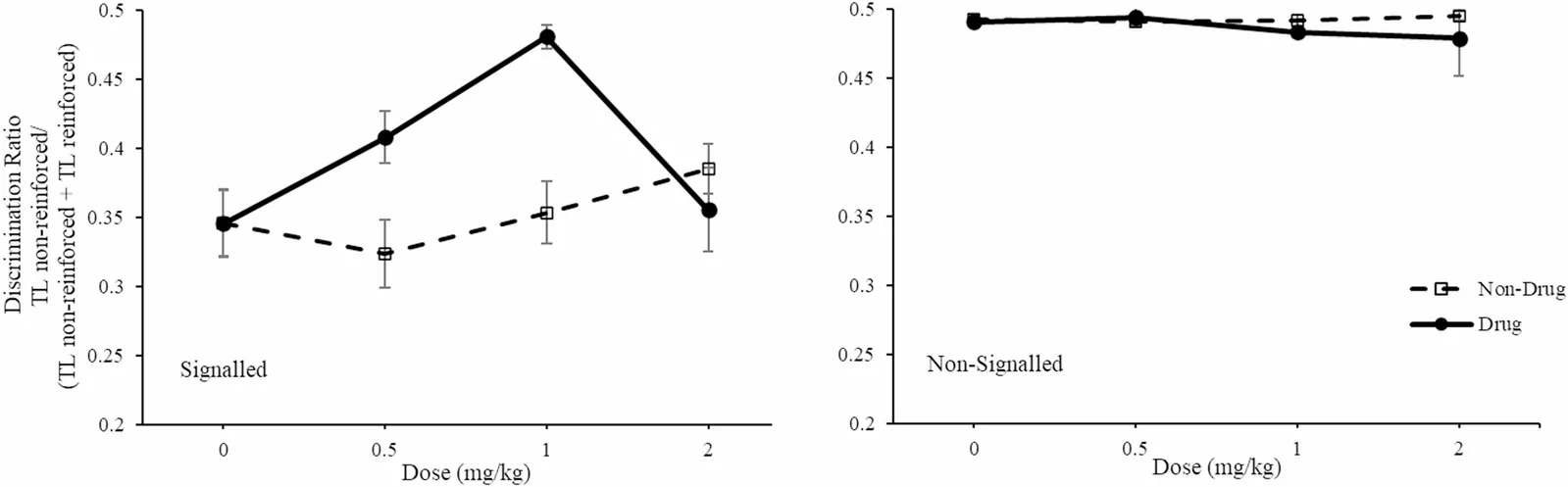
Discrimination ratios (TL non-reinforced/(TL non-reinforced + TL reinforced)) for Signalled (Left) and Non-Signalled (Right), Acute average drug and non-drug sessions at 0.0, 0.5, 1.0 and 2.0 mg/kg. Subjects 201–212. Bars indicate +/- 1 standard error (SE)

In order to determine how rats’ ability to respond in the terminal links depending on the signal for non-reinforcement in the acute and chronic drug administration phases, a discrimination ratio was calculated where the number of terminal-link responses in the non-reinforced trials were divided by the total of the non-reinforced and reinforced trials on the 0.5 alternative. Any value lower than 0.5 would indicate that subjects were able to infer the meaning of the signal and consequently reduce their responding in terminal links from which no reinforcement would be forthcoming. Average discrimination ratios are presented in Fig. 7. The 2.0 mg dose had inconsistent and decreased responding in the terminal links with sessions in which some rats responded only for a portion of the session or not at all, so results should be interpreted with caution. A linear mixed effects model with Signalled/Non-Signalled, Dose and Non-Drug/Drug as fixed effects, and a random intercept was applied to the Discrimination Ratio data. The intra-class correlation was significant, *r*_ICC_ = 0.288, *p* <.05, showing that the random effect accounted for 28.8% of the variance. There was a significant effect of Signalled/Non-Signalled, *F*(1, 161.118) = 220.578, *p* <.05, where Non-Signalled sessions (*M* = 0.490, 95% CI = 0.465, 0.514), had a significantly greater discrimination ratio than Signalled sessions (*M* = 0.371, 95% CI = 0.346, 0.396). Non-Drug/Drug just achieved a significant effect, *F*(1, 161.093) = 3.914, *p* =.05, where drug sessions (*M* = 0.438, 95% CI = 0.413, 0.463), had a significantly greater discrimination ratio than non-drug sessions (*M* = 0.422, 95% CI = 0.398, 0.447). There was a significant effect of Dose, *F*(3, 161.089) = 3.768, *p* <.05, a significant Signalled/Non-Signalled x Dose interaction, *F*(3, 161.113) = 4.398, *p* <.05, a significant Signalled/Non-Signalled x Non-Drug/Drug interaction, *F*(1, 161.118) = 7.771, *p* <.05 a significant Dose x Non-Drug/Drug interaction, *F*(3, 161.089) = 7.651, *p* <.05 and a significant Non-Signalled/Signalled x Dose x Non-Drug/Drug interaction, *F*(3, 161.113) = 6.014, *p* <.05. Simple Effects demonstrated that the 0.5 mg/kg, 1.0 mg/kg and 2.0 mg/kg Non-Drug discrimination ratios were not significantly different from the 0.0 mg/kg Non-Drug discrimination ratio in both the Signalled and Non-Signalled sessions. In the Drug Signalled sessions, the 0.5 mg/kg and 1.0 mg/kg doses had a significantly greater discrimination ratio than the 0.0 mg/kg dose. In the Signalled sessions, the 0.5 mg/kg and 1.0 mg/kg Drug sessions had a significantly greater discrimination ratio than the Non-Drug 0.5 mg/kg and 1.0 mg/kg sessions, whereas the 2.0 mg/kg Drug discrimination ratio was significantly smaller than the 2.0 mg/kg Non-Drug. The Signalled Non-Drug Discrimination Ratio had a significant linear contrast, *t*(3,161.027) = 2.105, *p* <.05. The Signalled Drug Discrimination Ratio had significant quadratic *t*(3,161.094)= −6.808, *p* <.05 and cubic *t*(3,161.041)= −3.381, *p* <.05 contrasts. There were no other significant effects.

These results indicate that in the signalled sessions, subjects responded differently when lack of forthcoming reinforcement was signalled at every drug dose compared to when it was not. The 0.5 mg/kg and 1.0 mg/kg drug doses had a higher discrimination ratio than the 0.0 mg/kg dose in the Signalled sessions and the 2.0 mg/kg drug dose had a smaller discrimination ratio than the 0.0 mg/kg dose in the Non-Signalled sessions. The 0.5 mg/kg and 1.0 mg/kg Signalled Drug discrimination ratios were also greater than the Non-Drug ratios and the 2.0 mg/kg Drug discrimination ratio was smaller than the Non-Drug ratio.

### Re-establish baseline

**Fig. 8:**
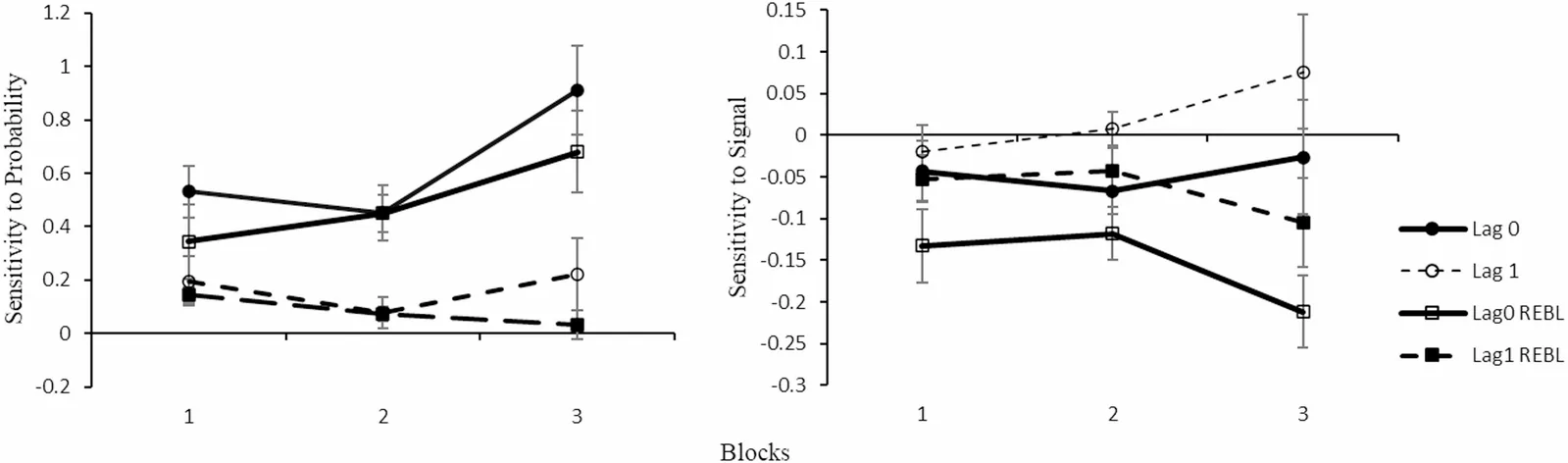
Lag Regression Analysis Results displaying average Sensitivity to Probability (Left) and Sensitivity to Signal (Right) of Baseline (BL) and Re-establish Baseline (RE-BL) across 18-trial blocks of a session. Subjects 201–212. Bars indicate +/- 1 standard error (SE)

Figure 8 displays the same lag regression analysis data as Fig. 3. with the addition of Lag0 (current session) and Lag 1 (previous session) data for the 16 re-establish baseline sessions immediately following the chronic phase. In these sessions, subjects received no methamphetamine administration. The data is averaged across the 12 subjects and the three, 18-trial blocks of each session. Best-fitting parameter estimates for Eq. 1 were obtained for each of the six session blocks for individual rats (overall average R^2^ = 0.315). A linear mixed effects model with lag, BL/RE-BL and block as fixed effects, and a random intercept was applied to the Sensitivity to Probability Lag0 and Lag 1 data. The intra-class correlation was significant, *r*_ICC_ = 0.192, *p* <.05, showing that the random effect accounted for 19.2% of the variance. There was a significant effect of Lag, *F*(1, 121) = 57.675, *p* <.05, where Sensitivity to Probability for Lag 0, (*M* = 0.561, 95% CI = 0.428, 0.694) was significantly greater than that of Lag 1, (*M* = 0.125, 95% CI= −0.009, 0.258). There was a significant effect of Block, *F*(2, 121) = 4.490, *p* <.05, and a significant Block x Lag interaction, *F*(2, 121) = 4.333, *p* <.05. Analysis of simple effects showed that Block 3 was significantly greater than Block 1 in both Baseline and Re-Establish Baseline, and Block 2 of Baseline. There was a significant linear component for both Lag 0 Baseline *t*(2,121) = 2.702, *p* <.05, and lag 0 Re-Establish Baseline *t*(2,121) = 2.3965, *p* <.05 and a quadratic component in Lag 0 Baseline, *t*(2,121)=−2.227, *p* < 05. There were no significant trends in Lag 1.

A linear mixed effects model with lag, BL/RE-BL and block as fixed effects, and a random intercept was applied to the Sensitivity to Signal Lag0 and Lag 1 data. The intra-class correlation was not significant, *r*_ICC_ = 0.064, *p* <.05. There was a significant effect of Lag, *F*(1, 121) = 10.391, *p* <.05, where Sensitivity to Signal for Lag 0, (*M*=−0.0999, 95% CI= −0.141, −0.059) was significantly smaller than that of Lag 1, (*M*=−0.023, 95% CI= −0.064, 0.019). There was a significant effect of Baseline/Re-Establish Baseline, *F*(1, 121) = 16.838, *p* <.05, where Baseline Sensitivity to Signal (*M*=−0.012, 95% CI= −0.054, 0.029), was significantly greater than Re-Establish Baseline Sensitivity to Signal (*M*=−0.110, 95% CI= −0.152, −0.069). There were no other significant effects.

### Response rates

**Fig. 9:**
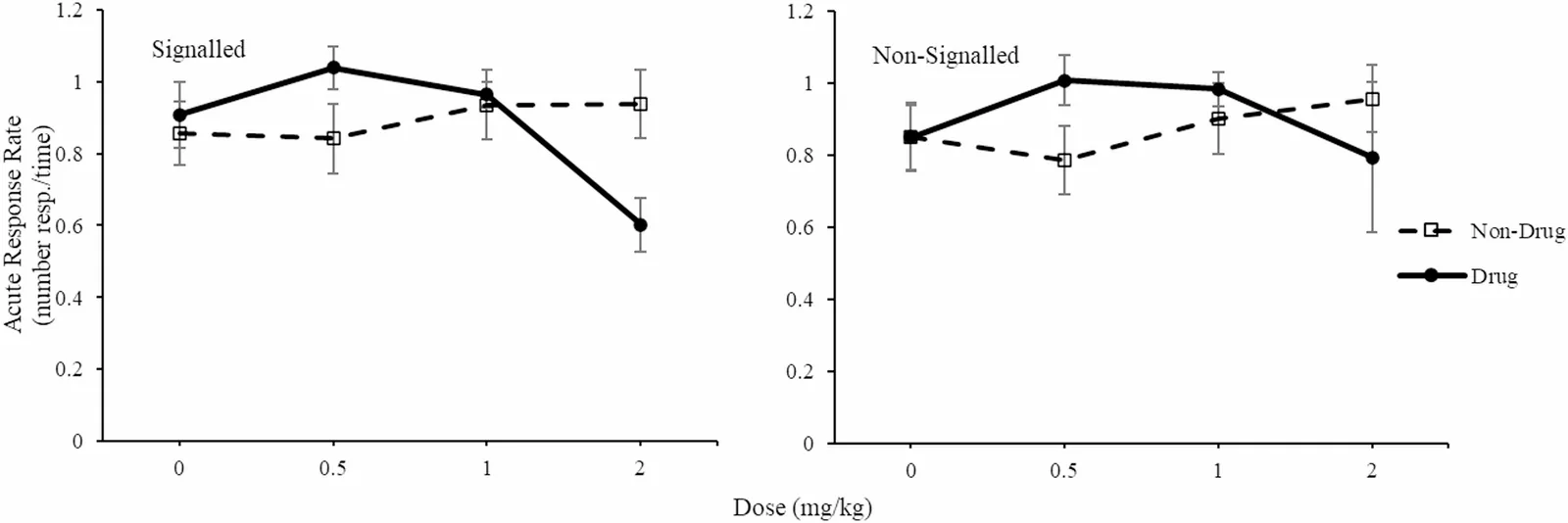
Average Signalled Sensitivity to Probability Response Rates (Left) and Non-Signalled Sensitivity to Probability Response Rates (Right). for Acute Non-Drug sessions and 0, 0.5, 1.0 and 2.0 mg/kg methamphetamine/Drug sessions. Subjects 201–212. Bars indicate +/- 1 standard error (SE)

Figure 9 displays the response rates for the acute signalled and non-signalled, non-drug and drug sessions. A linear mixed effects model with Signalled/Non-Signalled, Dose and Non-Drug/Drug as fixed effects and a random intercept was applied to the acute data. The intra-class correlation was significant, *r*_ICC_=0.617, *p* <.05, showing that the random effect accounted for 61.7% of the variance. There was a significant Dose x Non-Drug/Drug interaction, *F*(3,165) = 7.593, *p* <.05. Simple Effects revealed that for the 0.5 mg/kg dose, the drug response rate (*M* = 1.024, 95% CI = 0.841, 1.206) was significantly greater than the non-drug response rate (*M* = 0.814, 95% CI = 0.631, 0.997), and for the 2.0 mg/kg dose, the non-drug response rate, (*M* = 0.947, 95% CI = 0.765, 1.130) was significantly greater than the drug response rate, (*M* = 0.714, 95% CI = 0.531, 0.896. There were no other significant effects. These results demonstrate that there was a greater response rate at the 0.5 mg/kg dose for the drug sessions compared to the non-drug sessions, but a lower response rate for the drug sessions compared to the non-drug sessions at the 2.0 mg/kg dose.

**Fig. 10:**
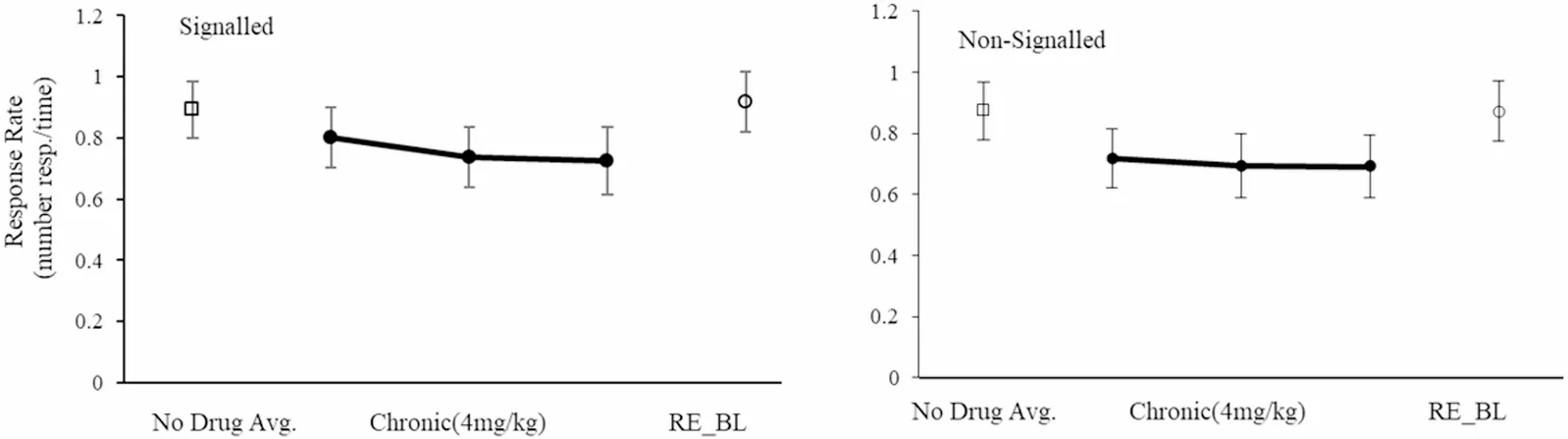
Average Signalled Sensitivity to Probability Response Rate (Left) and Non-Signalled Sensitivity to Probability Response Rate (Right). for combined Non-Drug Acute Average, Chronic Drug Phase and Re-Establish Baseline. Subjects 201–212. Bars indicate +/- 1 standard error (SE)

Figure 10 displays averaged Signalled (left) and Non-Signalled (Right), Response Rates for Non-Drug Average for the acute phase, three Chronic doses and the Re-Establish Baseline phase. A linear mixed effects model with Signalled/Non-Signalled and Drug Phase as fixed effects and a random intercept was applied to the acute data. The intra-class correlation was significant, *r*_ICC_=0.827, *p* <.05, showing that the random effect accounted for 82.7% of the variance. There was a significant effect of Drug Phase, *F*(4,99) = 10.365, *p* <.05. Post-Hoc Comparisons revealed that the No Drug Average Response Rate was significantly greater than the first Chronic Drug Dose, *t*(4,99) = 3.106, *p* <.05, the second Chronic Drug Dose, *t*(4,99) = 4.221, *p* <.05, and the third Chronic Drug Dose, *t*(4,99) = 4.389, *p* <.05. The Re-Establish Baseline Response Rate was also significantly greater than the first Chronic Drug Dose, *t*(4,99) = 3.402, *p* <.05, the second Chronic Drug Dose, *t*(4,99) = 4.517, *p* <.05, and the third Chronic Drug Dose, *t*(4,99) = 4.684, *p* <.05. There were no other significant effects. These results demonstrate that for both Signalled and Non-Signalled sessions, the response rates in the Chronic phase were lower than the acute Non-Drug average and the Re-establish Baseline.

## Discussion

We used a rapid acquisition, concurrent chains design to test rats’ sensitivity to probability alongside acute and chronic methamphetamine where in half of the sessions, outcomes were signalled. We found that in baseline, rats had a greater sensitivity to probability when the terminal-link outcomes (reinforcement or non-reinforcement) on the 50% alternative were differentially signalled than when they were not signalled. Rats could also effectively differentiate the current session from the previous one. In acute methamphetamine administration, sensitivity to probability decreased dose-dependently in signalled sessions, showing an increased propensity to the suboptimal choice. By contrast, acute methamphetamine had no effect on sensitivity to probability in un-signalled sessions.

In chronic drug administration, there were no effects of drug administration and sensitivity to probability remained greater in the signalled sessions than the un-signalled. In re-establish baseline, sensitivity to signal was smaller compared to that of baseline. Terminal link response rates demonstrated that in baseline and re-establish baseline, rats reduced their responding in signalled sessions when no reinforcement would be delivered at the conclusion of the terminal link. Discrimination ratios examined how rats responded in the acute drug phase when a lack of upcoming reinforcement was signalled or non-signalled. They responded significantly more when the lack of reinforcement was not signalled compared to when it was signalled. The 0.5 mg/kg and 1.0 mg/kg drug doses of the signalled sessions had a greater discrimination ratio than the signalled non-drug days, indicating that rats’ ability to respond differentially based on the cue for non-reinforcement was significantly impaired with 0.5 mg/kg and 1.0 mg/kg acute methamphetamine. The signalled 2 mg/kg dose would be expected to follow this trend, however, a decrease in discrimination ratio was reported. This was most likely caused by the lack of consistent responding in the terminal links of the 2 mg/kg drug administration sessions. Overall response rates showed that acute drug sessions had a greater response rate than non-drug sessions, the 2.0 mg/kg drug administration in non-signalled sessions had a greater response rate than all other doses, and the acute non-drug average and re-establish baseline had greater response rates than all three chronic doses in both signalled and non-signalled sessions.

The signalling effect in probabilistic choice is an often-studied phenomena with pigeons because it is a striking instance of suboptimal behaviour. It has been proposed as an important analogue of human gambling which can become an addiction despite net losses (Molet et al. 2012; Stagner and Zentall 2010; Zentall 2023). From a neurochemical perspective, gambling involves similar neurotransmitters and brain structures as stimulant drugs of addiction, but with gambling, these occur purely from conditioned external reinforcers as opposed to a chemical that is introduced into the body that directly influences levels of neurotransmitter release or reuptake (Zack and Poulos 2009). This provides a rationale for translational studies such as the present research which examines the effects of stimulant drugs on analogue behavioural tasks for gambling.

However, unlike pigeons, the rats in our study did not behave suboptimally, rather, they demonstrated a greater preference for the 100% alternative, in the presence of the signal compared to when there was no signal. An explanation for this may be found by examining the opposite behaviour, why pigeons behave suboptimally in the presence of a signal. Zentall (2014) suggests that conditioned reinforcers that inform pigeons that reinforcement will occur exert more control over responding than conditioned inhibitors that inform pigeons that reinforcement will not occur.

Perhaps in our study, the opposite effect was occurring, in that the light and tone that informed the rat that reinforcement would not occur after 30 s, exerted more control than the flashing lever light that informed them that reinforcement would occur. Thus, the signal that indicated no forthcoming food on the 50% alternative became a conditioned inhibitor, which resulted in less suboptimal choice on the signalled sessions than the non-signalled. Trujano et al. (2016) found that rats preferred the optimal alternative, even though it was preceded by non-differential stimuli over the suboptimal alternative that was preceded by discriminative stimuli. Therefore, rats appeared to be influenced to a greater extent by the signal for non-reinforcement than that for reinforcement and this influence does not lessen as a result of training.

Zentall (2022) discussed pigeon’s suboptimal behaviour in terms of contrasts based on perceived losses and gains. He explains that in an experiment with the same reinforcer rates as ours, pigeons would prefer the 50% alternative because the signal for reinforcement on this option produces a positive contrast greater than the expected reinforcement on the alternative which is always 100% meaning that no contrast occurs. Similar to conditioned inhibition, the opposite could be said for our experiment, where the loss of reinforcement on the signalled 50% alternative might have produced a greater negative contrast than the gains achieved from positive contrast on the signalled alternative, and this drove responding toward the 100% alternative in the signalled sessions.

Chow et al. (2017) investigated how the different types of signals that are presented during the terminal link may invoke different levels of preference for reinforcement in rats. They reported that preference for the signalled alternative was greater with a lever cue compared to a light cue. They concluded that when value is attributed to a terminal link stimulus, it controls preference to a greater extent than the reinforcer. This may have had an influence in the results of our procedure, as even though subjects responded on levers, they were present both in signalled and non-signalled conditions and at both probabilities of reinforcement. So unlike in the Chow et al. (2017) procedure, value would not be attributed to them because they were not indicative of reinforcement. The signal that did indicate forthcoming reinforcement, the flashing lever light, did not have enough value attributed to it to overtake the probability of reinforcement and promote preference for the suboptimal alternative. This may have occurred because the location of the signal for upcoming reinforcement in 50% signalled trials, changed unpredictably between sessions in the rapid acquisition design. Sometimes the lever light that signalled reinforcement was located above the left lever, and sometimes it was above the right. In contrast, the flashing house light and tone that signalled lack of reinforcement did not change location between sessions, meaning it may have been more salient than the signal for reinforcement, and so exerted a greater influence than the lever light resulting in an increased preference for the certain alternative.

Perhaps the amount of time that the subject is in the presence of the signal is important and this then influences how behaviour is allocated (Cunningham and Shahan 2019). In Roper and Baldwin’s (2004) third experiment, preference for the discriminative alternative developed when the signal length was increased from six seconds to 24 s. However, our terminal links were FI 30 s, but preference for the sub-optimal alternative did not develop and was even less on signalled compared to non-signalled sessions.

This result becomes more interesting in the light of the influence of methamphetamine in our procedure. Increasing doses of acute methamphetamine dose dependently decreased preference for the 100% reinforcer, but only when the outcome was signalled. When the outcome was not signalled, methamphetamine produced no difference in responding compared to the non-drug sessions. This is opposite to Bodeker and Grace (2024) results, where the same methamphetamine administration regimen reduced sensitivity to probability in the absence of any signal in the terminal link. However, in that procedure, the terminal link length was only 2.83 s whereas it was increased in the current study to ensure adequate exposure to the discriminative stimuli in the signalled sessions. Therefore, the lack of difference in responding between the acute drug sessions and the non-drug sessions in the non-signalled sessions only, resulted either from the increased terminal link length or from the concurrent exposure to signalled sessions. The discrimination ratios, (which indicated response rates in the terminal links when reinforcement was not forthcoming) in the acute drug phase were significantly greater than the non-drug sessions during the 0.5 mg/kg and 1.0 mg/kg doses in the signalled sessions. This may suggest that the methamphetamine disrupted the interpretation of the signal which caused rats to increase response rates just as they did in the non-signalled sessions when they did not know if reinforcement would occur. Further research would need to be conducted, perhaps with a variety of terminal link lengths to elucidate this interesting difference in preference allocation and responding under stimulant administration.

Also contrary to Bodeker and Grace (2024), overall response rates in the current study, were initially greater in both the signalled and non-signalled acute drug administration sessions compared to the non-drug sessions, as opposed to uniformly reducing during acute drug administration sessions. This may better reflect the influence of methamphetamine that can be observed in human studies and suggests that longer terminal links may provide a better animal model for human choice (Hart et al. 2012).

The remaining differing factor of our procedure was the rapid acquisition design where one of four possible session types occurred pseudo-randomly each day and subjects had to discern which session type was in effect at the start of each session. In contrast to this, most sub-optimal choice procedures have forced trials (Zentall and Stagner 2011; Trujano and Orduña 2015; Cunningham and Shahan 2019) at the beginning of a session in which only one of the two alternatives is presented in order to demonstrate the outcome of each alternative to the subject before choice trials begin. However, McDevitt et al. (2022) suggest that these forced exposure trials may be enhancing suboptimal choice behaviour. They reported that the groups that had forced exposure trials chose suboptimally in the choice trials whereas the group that only ever had both alternatives presented, displayed a range of behaviours from extreme optimal choice to extreme suboptimal. The authors suggest that this wide range of choice may have because of inadequate sampling, meaning that if a pigeon developed a bias for or against one of the alternatives early on in training, that would then govern their behaviour for the remainder of the sessions. Forced exposure can remedy this by ensuring that adequate exposure to the outcomes of both alternatives is experienced. The procedure of the current experiment overcame this risk while maintaining the presentation of both alternatives, which may be advantageous as it allows the consideration of one alternative within the context of the other, by assigning reinforcers to each of the alternatives equally. This meant that rats were not truly choosing between the signalled suboptimal and non-signalled optimal levers, but rather they indicated their preference during the initial links and only gained entry to the terminal links by responding on the lever to which the reinforcer had been allocated. This ensured that rats experienced the consequences of both alternatives equally but still demonstrated which they preferred by their initial link response rates.

Few other studies examine the influence of gambling paradigms in animals alongside stimulant administration, but Ferland et al. (2019) reported on their procedure where rats trained on a risky choice task where outcomes were cued or un-cued, before self-administering cocaine in conjunction with completing the task. They found that rats that had trained on the cued task were more likely to make risky choices, and that those rats displayed even more risky choice following cocaine administration compared to those that had trained on the un-cued task. This suggests a role for reliably signalled reinforcement to perpetuate addiction, particularly in subjects with an underlying propensity to impulsivity. These results may also relate to our findings where methamphetamine only resulted in a reduced sensitivity to probability in signalled sessions.

Our experiment, where rats responded in rapid acquisition concurrent chains where uncertain reinforcement was sometimes signalled under methamphetamine administration resulted in a number of interesting findings. Rats preferred the optimal option to a greater extent in the presence of the signal than in its absence. Acute methamphetamine dose dependently decreased sensitivity to probability and increased preference for the uncertain alternative but only in the signalled sessions. Response rates increased in the presence of 0.5 mg/kg and 1.0 mg/kg cute methamphetamine, but decreased under chronic administration. Our findings are limited by a number of factors, a highly constrained laboratory setting cannot replicate the complexity of influences incumbent on human choice behaviour and although it is advantageous to examine discrete aspects, the interpretation of our results cannot be applied broadly. Zentall’s (2023) review for example, reported that motivational level and environmental enrichment impacted the degree to which pigeons behaved suboptimally. Our experimental design may have precluded the observation of changes of preference, particularly in the chronic phase where repeated methamphetamine administration may have produced a change that could not be observed because the experimental contingencies changed unpredictably across sessions.

As discussed above, the signals that preceded reinforcement or non-reinforcement outcomes may not have been as salient for rats as for pigeons in comparable research. In order to make those stimuli more salient for rats we did not counterbalance the flashing lever light and houselight/tone as signals for reinforcement or non-reinforcement. In this way, the location of the flashing lever light covaried with the signalled outcome across sessions. In future research, it would be interesting to counterbalance this assignment to determine if the signalling effect was independent of the stimulus modality. Nevertheless, results of our experiment provide a novel contribution to research on signalled choice, and the rapid-acquisition concurrent chains procedure may prove useful for future investigation with animal models of gambling.

## Supplementary Information

Below is the link to the electronic supplementary material.

Supplementary Material 1 (PDF 161 KB)

## Data Availability

Data is available on request from Rebecca Bodeker at bodekerrebecca@gmail.com.
