# Supplementary material for: Effects of methamphetamine on signalled probability in rats using concurrent chains
Source: Psychopharmacology (Berl). 2026 Jan 23;243(11):2523–37. doi: 10.1007/s00213-026-07006-x (PMC13710648; doi:10.1007/s00213-026-07006-x)
Supplement: Supplementary file 1 [file 213_2026_7006_MOESM1_ESM.pdf]

5. SUPPLEMENTARY MATERIALS

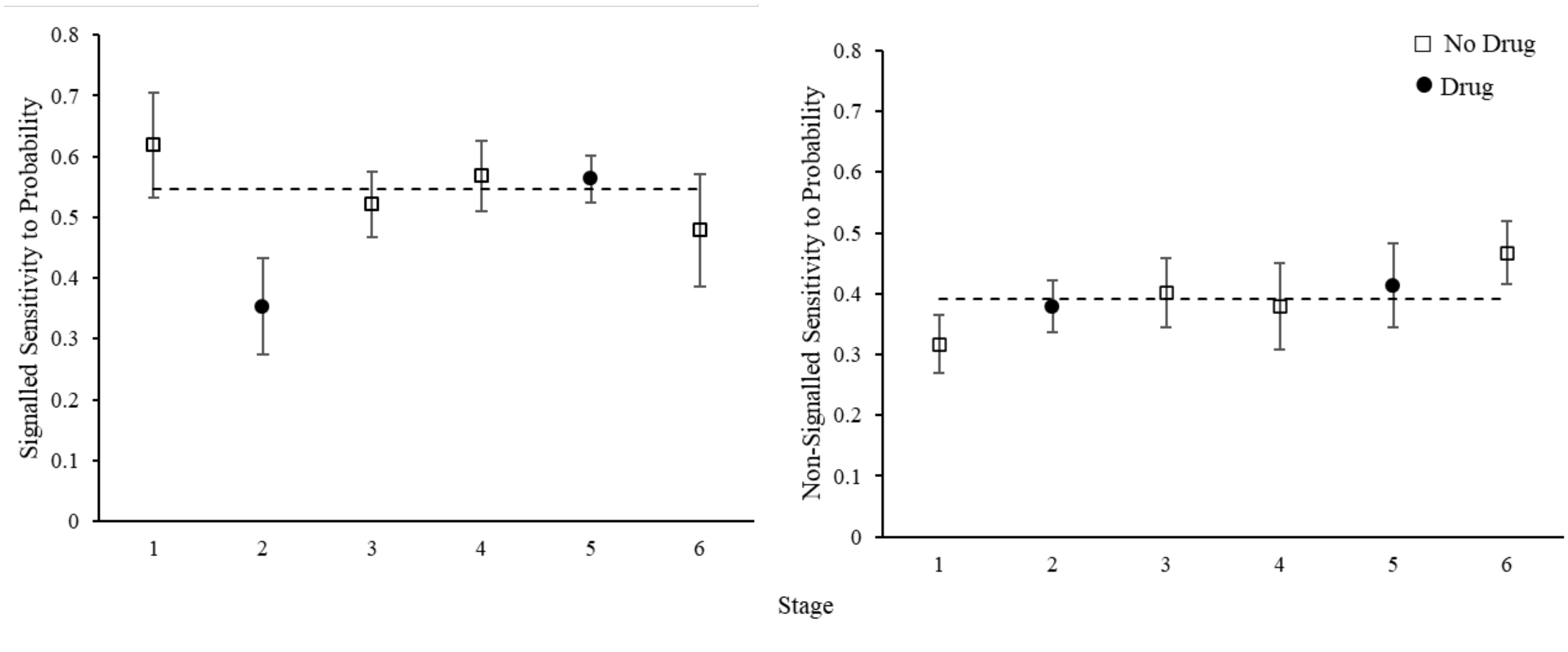

Figure 11. Average control and 0.0mg/kg (saline) dose effect sizes for Signalled Sensitivity to Probability (Left) and Non-Signalled Sensitivity to Probability (Right). Saline was administered for stages 2 and 5. Subjects 201-212. Bars indicate +/- 1 standard error (SE). Dashed line indicates average of No Drug Stages.

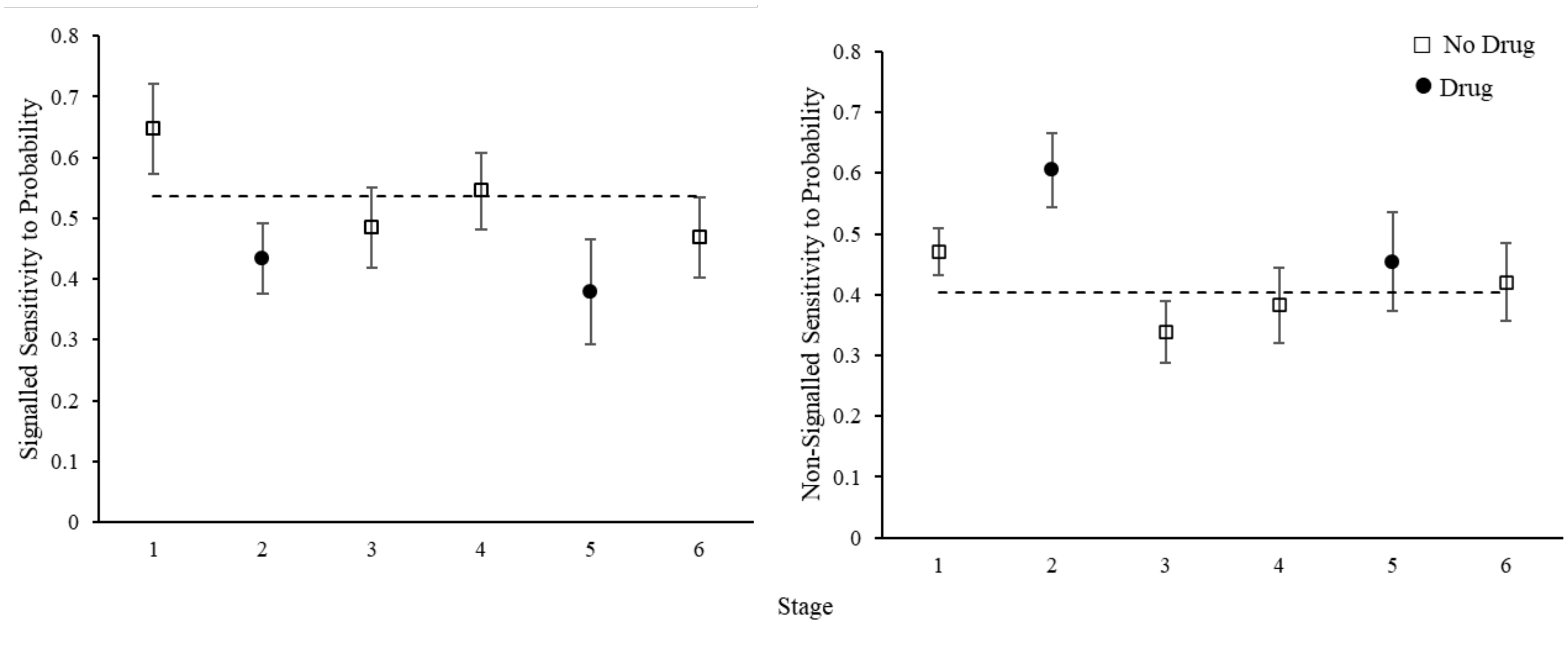

Figure 12. Average control and 0.5mg/kg methamphetamine dose effect sizes for Signalled Sensitivity to Probability (Left) and Non-Signalled Sensitivity to Probability (Right). Saline was administered for stages 2 and 5. Subjects 201-212. Bars indicate +/- 1 standard error (SE). Dashed line indicates average of No Drug Stages.

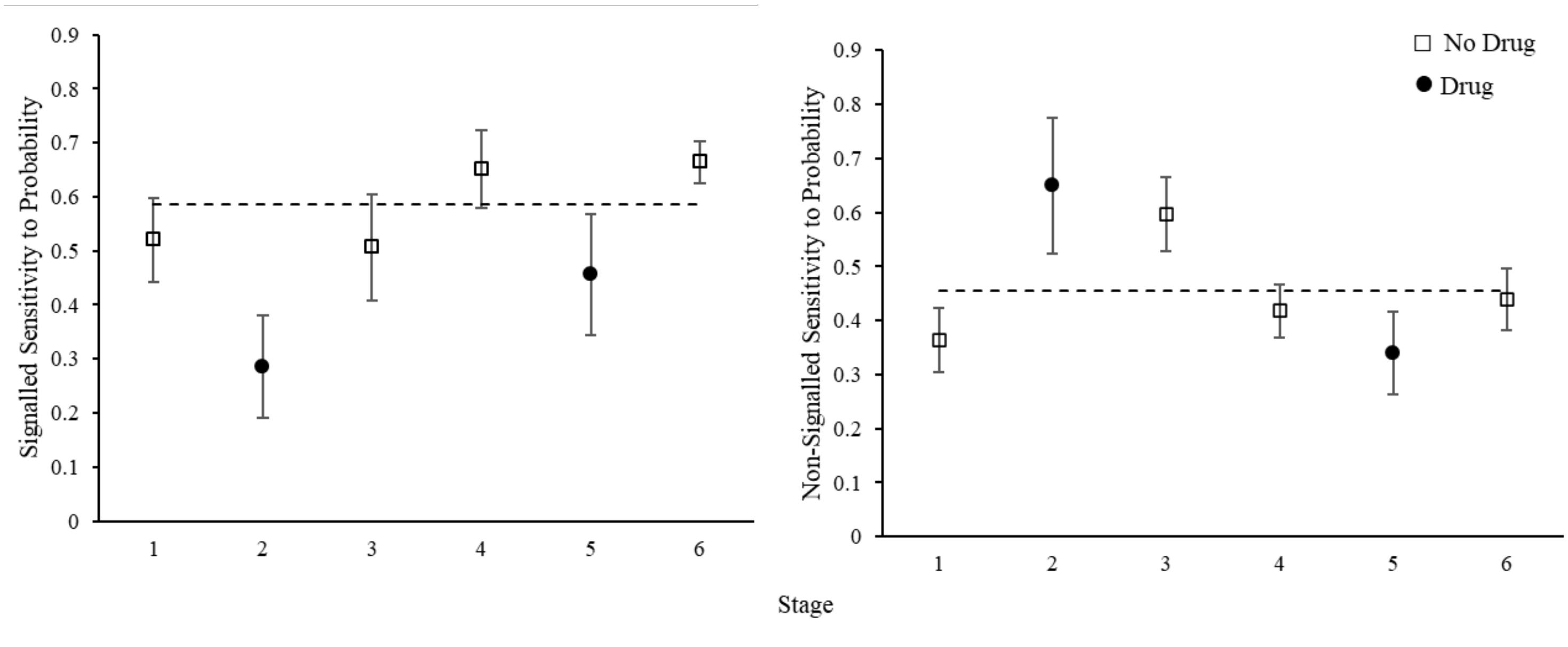

Figure 13. Average control and 1.0mg/kg methamphetamine dose effect sizes for Signalled Sensitivity to Probability (Left) and Non-Signalled Sensitivity to Probability (Right). Saline was administered for stages 2 and 5. Subjects 201-212. Bars indicate +/- 1 standard error (SE). Dashed line indicates average of No Drug Stages.

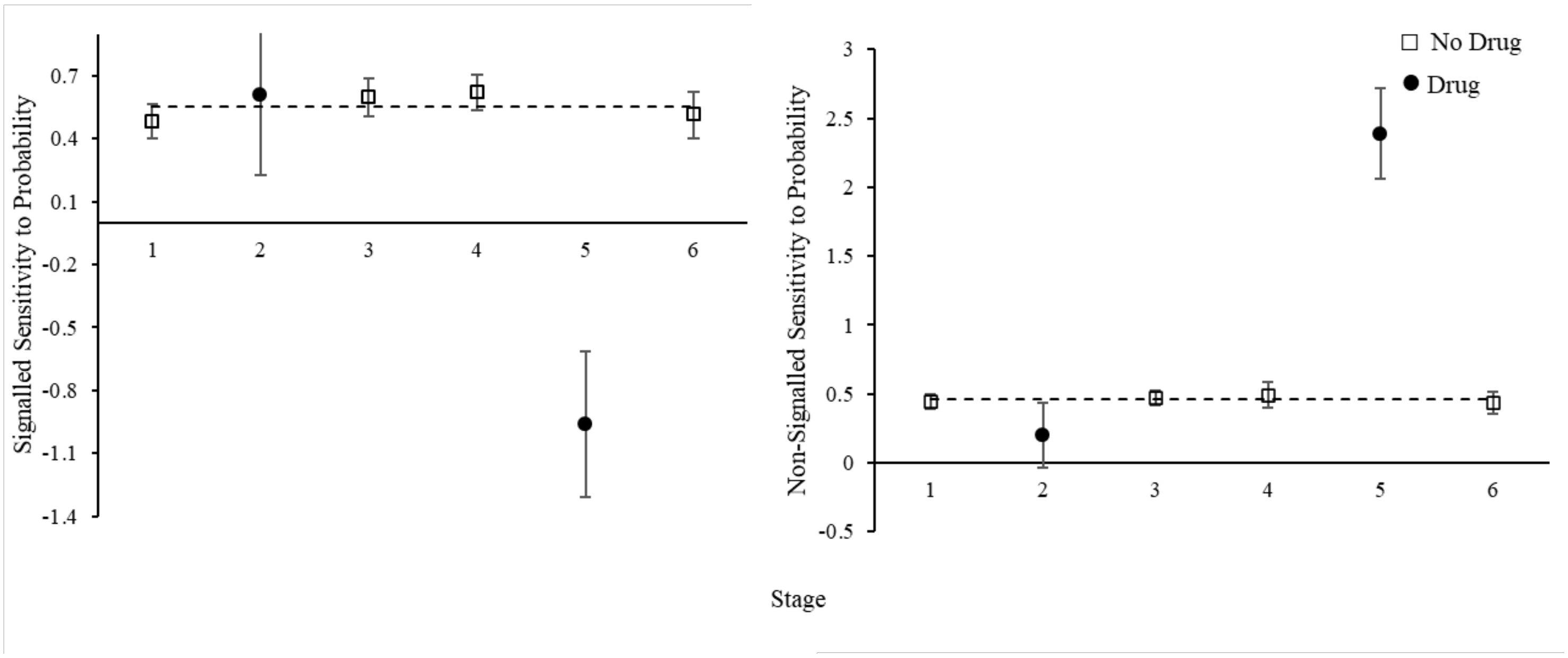

Figure 14. Average control and 2.0mg/kg methamphetamine dose effect sizes for Signalled Sensitivity to Probability (Left) and Non-Signalled Sensitivity to Probability (Right). Saline was administered for stages 2 and 5. Subjects 201-212. Bars indicate +/- 1 standard error (SE). Dashed line indicates average of No Drug Stages.

After baseline training, the acute phase was run for 96 consecutive sessions. Each of the four methamphetamine doses (0.0, 0.5, 1.0 and 2.0mg/kg), consisted of 24 sessions arranged in such a way that a drug administration day would be followed by two non-drug days. Twelve sessions measured Signalled Probability and twelve measured Non-Signalled Probability resulting in six stages of each effect size calculation. Within those twelve sessions, eight days were non-drug sessions and four were drug sessions. Figures 11-14 display the Signalled Probability (left) and Non-Signalled Probability Effects (right) for the separate drug doses.
